# Detection of sleep disordered breathing severity using acoustic biomarker and machine learning techniques

**DOI:** 10.1186/s12938-018-0448-x

**Published:** 2018-02-01

**Authors:** Taehoon Kim, Jeong-Whun Kim, Kyogu Lee

**Affiliations:** 1 Music and Audio Research Group, Graduate School of Convergence Science and Technology, Seoul National University, 1 Gwanak-ro, Seoul, 08826 Republic of Korea; 2 Department of Otorhinolaryngology, Seoul National University Bundang Hospital, Seoul National University College of Medicine, Gumi-ro, Seongnam, 13620 Republic of Korea

**Keywords:** Sleep disordered breathing, Acoustic biomarker, Deep neural network, Polysomnography screening test, Apnea–hypopnea index

## Abstract

**Purpose:**

Breathing sounds during sleep are altered and characterized by various acoustic specificities in patients with sleep disordered breathing (SDB). This study aimed to identify acoustic biomarkers indicative of the severity of SDB by analyzing the breathing sounds collected from a large number of subjects during entire overnight sleep.

**Methods:**

The participants were patients who presented at a sleep center with snoring or cessation of breathing during sleep. They were subjected to full-night polysomnography (PSG) during which the breathing sound was recorded using a microphone. Then, audio features were extracted and a group of features differing significantly between different SDB severity groups was selected as a potential acoustic biomarker. To assess the validity of the acoustic biomarker, classification tasks were performed using several machine learning techniques. Based on the apnea–hypopnea index of the subjects, four-group classification and binary classification were performed.

**Results:**

Using tenfold cross validation, we achieved an accuracy of 88.3% in the four-group classification and an accuracy of 92.5% in the binary classification. Experimental evaluation demonstrated that the models trained on the proposed acoustic biomarkers can be used to estimate the severity of SDB.

**Conclusions:**

Acoustic biomarkers may be useful to accurately predict the severity of SDB based on the patient’s breathing sounds during sleep, without conducting attended full-night PSG. This study implies that any device with a microphone, such as a smartphone, could be potentially utilized outside specialized facilities as a screening tool for detecting SDB.

## Background

Sleep disordered breathing (SDB) is characterized by repetitive episodes of partial or complete obstruction of the upper airway during sleep. This disease is frequently observed in patients with hypertension, cardiac arrhythmia, coronary artery disease, stroke, and type 2 diabetes. It has also been revealed as an independent risk factor for these disorders. Even though the prevalence of SDB is as high as 24% in men and 9% in women, based on the apnea–hypopnea index (AHI) criterion of ≥ 5/h, SDB remains under-recognized and underdiagnosed [1]. Although polysomnography (PSG) is a standard examination for diagnosing SDB, it is subjected to several limitations. These include high cost, insufficient capacity for examination compared to the large number of patients, and the first night effect leading to reduced sleep efficiency and quality [2–5]. Because of these limitations, assessing the treatment outcome is difficult. Home screening can be useful to address these problems, and ambulatory sleep monitoring devices are used for this purpose. However, all the devices used in practice are placed on the patient’s body and may lead to patient discomfort due to the recording equipment.

Previous studies have analyzed snoring sounds occurring during sleep and identified their relationship with AHI, as snoring is a common symptom of SDB [6–12]. In addition to snoring sounds, various abnormalities of the sound per se, or the sound pattern, are observed in patients with SDB [13]. Respiratory sounds during inhalation and exhalation may be noisy when the upper airway space is compromised. Breathing intervals and patterns during sleep are also disturbed. Irregular breathing sounds will be detected when partial or complete breathing cessations occur due to hypopnea or apnea. Breath holding, breathing interruptions, gasping, and choking may also be detected [14].

Considering the acoustic properties of SDB, many studies have investigated the relationships between audio features and pathological symptoms or AHI scores. Common approaches for the analysis of sleep sound are physical measurements of the sound strength such as frequency spectrum, weighted sound intensity, and root mean square value of a signal [15]. Alternatively, different approaches such as the analysis of inter-SDB event silence and identification of snoring irregularity have also been attempted. Ben-Israel et al. found that the inter-event silence, which is associated with the acoustic energy patterns of SDB events, was significantly correlated with AHI [16]. They also estimated AHI using a multivariate linear regression model and found that the estimated AHI correlated with AHI measured on PSG. Another approach is to tailor the feature extraction process using signal patterns on the temporal or spectral domain. Mesquita et al. proposed a method identifying two distinct types of snoring: non-regular and regular snoring [17]. They extracted snoring sounds from respiratory sound signals and analyzed time intervals between regular snoring sounds in short segments of overnight recordings. They found that subjects with severe SDB had a shorter time interval between regular snoring and less dispersion on the time interval features. However, many recent studies have adopted the use of event detectors for estimation of snoring events [8, 18–23]; such methods may have disadvantages when using noisy data, leading to low detection performance and a decrease in system robustness.

In our study, we aimed to extract an acoustic biomarker from breathing sounds recorded during the entire overnight sleep that would allow us to classify the severity of SDB in patients using deep neural network. The acoustic biomarker consists of several audio features that describe the acoustic characteristics of the patient’s breathing sound during sleep. We hypothesized that breathing sounds may carry important information, and thus, by analyzing such audio signals obtained from a large number of subjects, it may be possible to identify acoustic biomarkers that indicate the severity of SDB. Thus, we collected breathing sound data from full-night PSG and extracted several standard audio features, as well as audio features used in sleep sound analysis. We then used statistical analysis to identify acoustic biomarkers and verified their effectiveness using a train-test classification framework based on the SDB severity.

## Methods

### Subjects

The patients who visited a Seoul National University Bundang Hospital (SNUBH) sleep center between October 2013 and March 2014, because of snoring or cessation of breathing during sleep, were recruited as subjects. The subjects underwent a full-night PSG (Embla^®^ N7000, Natus neurology). We classified the subjects into four SDB severity groups according to their AHI: normal (AHI < 5), mild (5 ≤ AHI < 15), moderate (15 ≤ AHI < 30), and severe (AHI ≥ 30). Each group consisted of 30 patients, with a total of 120 patients included the study. Among the 120 subjects, there were 3 children (age < 8) and 4 adolescents (8 ≤ age < 18).

### Acquisition of sleep sounds

**Fig. 1:**
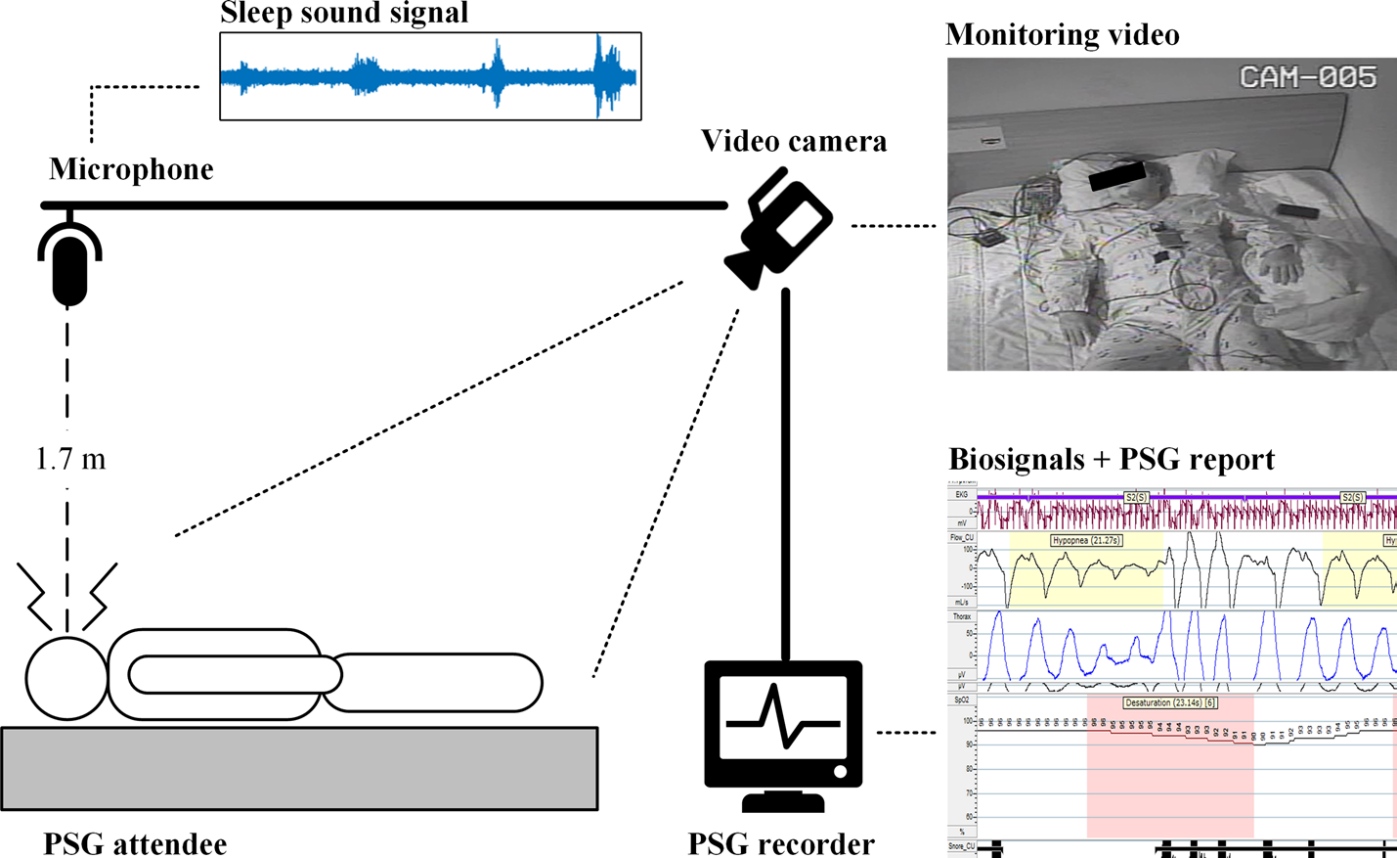
Acquisition of sleep sounds and PSG reports in sleep laboratory. Audio data and PSG reports were recorded from the PSG system. After acquisition, two filtering stages were adopted to eliminate unwanted noises for 120 patients

All the subjects underwent an attended full-night PSG in the sleep laboratory of the SNUBH, South Korea. All procedures performed on human participants were in accordance with the ethical standards of the Institutional Review Boards at the SNUBH (IRB-B-1404/248-109). The breathing sound during sleep was recorded as a part of the PSG using a PSG-embedded microphone (SUPR-102, ShenZhen YIANDA Electronics Co. Ltd., Shenzhen, China) placed on the ceiling above the patient’s bed, at a distance of 1.7 m as shown in Fig. 1. Since the microphone was used for recording environmental sounds during PSG, we used breathing sounds contained within the environmental sounds as an input signal. The sampling frequency of the recordings was 8 kHz. No additional analog filters were applied for signal correction, and raw recordings were used for the study. The mean recording time was 7 h 10 m 30 s.

### Preprocessing

The recorded audio contained sounds from various sources of noise (e.g. PSG machine noise, duvet noise, conversation between a clinician and a patient) in addition to the target sleep sound. Our study aimed to extract the acoustic marker representing the severity of SDB; the noises not related to breathing mentioned above can create problems during the analysis of features extracted from recorded sleep sounds. Overcoming this problem required the use of several sound filtering processes to distinguish the meaningful sleep sounds from unwanted noise.

To this end, we performed a two-stage filtering process to remove various unwanted noises and purify the sleep breathing sounds. First, we filtered breathing sounds using spectral subtraction filtering method [24] given that the spectra of noises does not change the target signal and that subtractive filtering method is computationally efficient [25]. We also applied sleep stage filtering to eliminate the noises originating from conversations and the sound of duvet. During stages 2 and 3 non-REM (rapid eye movement) sleeps, that comprise most of the sleep [26], the respiration is quite stable and regular compared to stage 1 and REM sleeps. Also, muscle activity during the REM and stage 1 sleeps results into unintended noises such as the duvet noise [9]. Therefore, we focused on sleep breathing sounds occurring during stages 2 and 3 sleeps. The mean length of filtered sounds was 4 h 1 m 55 s, which is 56.19% of the mean recording time.

### Extraction of audio features

**Fig. 2:**
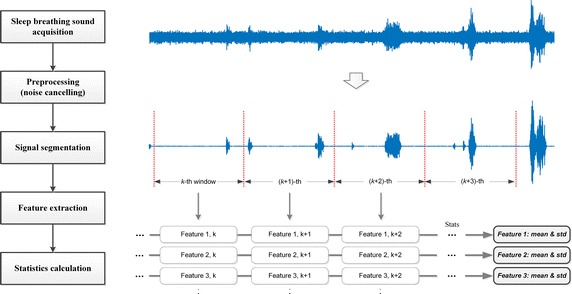
Audio feature extraction framework. Audio features were extracted in every 5 s windows. Then statistical values (means and standard deviations) of features were calculated over whole sleep period

Several audio features were extracted from the preprocessed signals to estimate the severity of SDB. These audio features included the mel frequency cepstral coefficients (MFCCs), spectral flux, and zero crossing rate, and represented a variety of temporal and spectral characteristics of an audio signal. Figure 2 outlines the overall feature extraction process used in this study.

The whole-night sleep breathing sound was divided into multiple windowed signals. A window size of 5 s was adopted, and the above-mentioned audio features were then extracted from each windowed signal. This gave a sequence of 5760 values per each audio feature, assuming an 8-h-long sleep period. We calculated the mean and standard deviation of the values for all features as their representative values. Table 1 shows the list of extracted features and their descriptions according to the feature extraction framework. All features were extracted with jAudio (java-based audio feature extraction software), and statistical analyses were performed using MATLAB R2016a (MathWorks, Inc., MA, USA).

**Table 1:** List of extracted audio features

Feature (abbreviation)	Description	# of variables
Spectral centroid (SC)	Center of mass of the spectrum	1
Spectral rolloff point (SR)	Right skewness of the power spectrum	1
Spectral flux (SF)	Amount of spectral change of the signal	1
Compactness	Sum of results of fast Fourier transform over frequency bins	1
Spectral variability (SV)	Variance of the magnitude spectrum	1
Root mean square (RMS)	Power of the signal	1
Fraction of low energy windows (FLEW)	Quietness of the signal relative to the rest of the signal	1
Zero crossings (ZC)	The number of times the signal changes sign from one sample to another	1
Strongest beat (SB)	Highest bin in the beat histogram	1
Beat sum (BS)	Sum of all values in the beat histogram	1
Strength of strongest beat (SSB)	Strength of the strongest beat in the signal	1
Strongest frequency via ZC (SF-ZC)	Strongest frequency in the signal by looking at the ZC	1
Strongest frequency via SC (SF-SC)	Strongest frequency in the signal by looking at the SC	1
Strongest frequency via FFT max (SF-FFT)	Highest bin in the power spectrum	1
MFCC	Short-term power spectrum based on the nonlinear mel scale of frequency	13 (0–12)
Constant-Q based MFCC (CQ-MFCC)	MFCC that directly calculates the logarithmic frequency bins	
Linear predictive coding (LPC)	Spectral envelope based on the information of a linear predictive model	10 (0–9)
Method of moments (MM)	Calculation of the first 5 statistical method of moments	5 (0–4)
Relative difference function (RDF)	Log of the derivative of the RMS	1
Area method of moments (AMoM)	Numeric quantities at some distance from a reference point or axis	10 (0–9)
AMoM of MFCC	AMoM derived with MFCC values instead of the density distribution function	10 (0–9)
AMoM of CQ-MFCC	AMoM derived with CQ-MFCC values instead of the density distribution function	10 (0–9)
AMoM of Log of CQ Transform (LCQT)	AMoM derived with Log Constant-Q Transform values instead of the density distribution function	10 (0–9)

In addition, other audio features that were demonstrated as effective for sleep sound analysis in previous studies [15, 27, 28] were adopted for analysis. Some of these features were added to the extraction framework as detailed below.

First, the formants of sleep breathing sounds were extracted. We extracted the amplitudes of the initial three peaks in the frequency domain (F1, F2, and F3 formants) from overnight sleep breathing sounds, and calculated the maximum, minimum, mean, and standard deviation of the formants of each patient across the windows. Second, we computed a sub-band energy distribution of the sleep breathing sound, which is the energy distribution of a given signal among partitioned frequency bands. The formants and sub-band energy distribution are both useful to distinguish snoring events from non-snoring events. A Gammatone filter bank, a widely used method for auditory signal processing, was applied for sub-band analysis, as it reflects the frequency analysis of the human ear. Likewise, simple statistics of the sub-band energy distribution, such as maximum, minimum, mean, and standard deviation, were calculated and regarded as candidate features. The weighted sound intensities, which describe the loudness of a sound as perceived by a human, were computed to function as another extra audio feature. This computation used various sound pressure levels (SPLs), such as the A-weighted sound pressure level (dBA), the C-weighted sound pressure level (dBC), and the linear-weighted (unweighted) sound pressure level (dB). The A-weighting scale was modeled to reflect the frequency sensitivity of the human ear and the C-weighting scale mimicked the frequency sensitivity of the human ear within a loud noise environment.

### SDB severity group discriminators and the acoustic biomarker

**Fig. 3:**
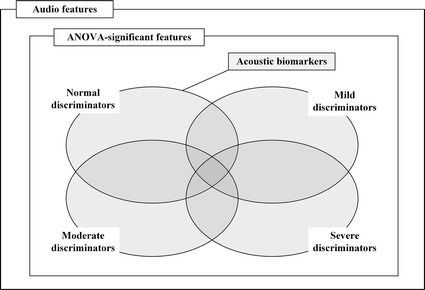
Selection of acoustic biomarker. Among statistical values of audio features, SDB-severity group discriminators were determined with Tukey-HSD tests. The union of all SDB-severity group discriminators is defined as the acoustic biomarker

To identify an acoustic biomarker, we first defined a concept of SDB severity group discriminators. A one-way ANOVA test was performed on the extracted audio features to evaluate significant differences across the SDB severity groups. A Tukey honest significant difference (HSD) test was used to evaluate significant differences between each pair of SDB severity groups, and to identify group discriminators. We assumed that features with *p* value < 0.05 can be used to distinguish among the SDB severity groups. The SDB severity group discriminators were the features that could statistically differentiate between a specific SDB severity group and the other groups. For example, features showing a statistical significance between the normal and other groups (mild, moderate, and severe) were defined as normal group discriminators. Other severity group discriminators were also defined in the same way. Consequently, the collection of discriminators for the four groups (normal, mild, moderate, and severe SDB severity) was defined as the acoustic biomarker. That is, the acoustic marker is a set of statistically significant features that can discriminate among the SDB severity groups. Figure 3 shows relations between audio features and the acoustic biomarker. We also applied the feature selection algorithm based on SVM to acoustic biomarker and selected.

### Extraction of quantized transition matrix

**Fig. 4:**
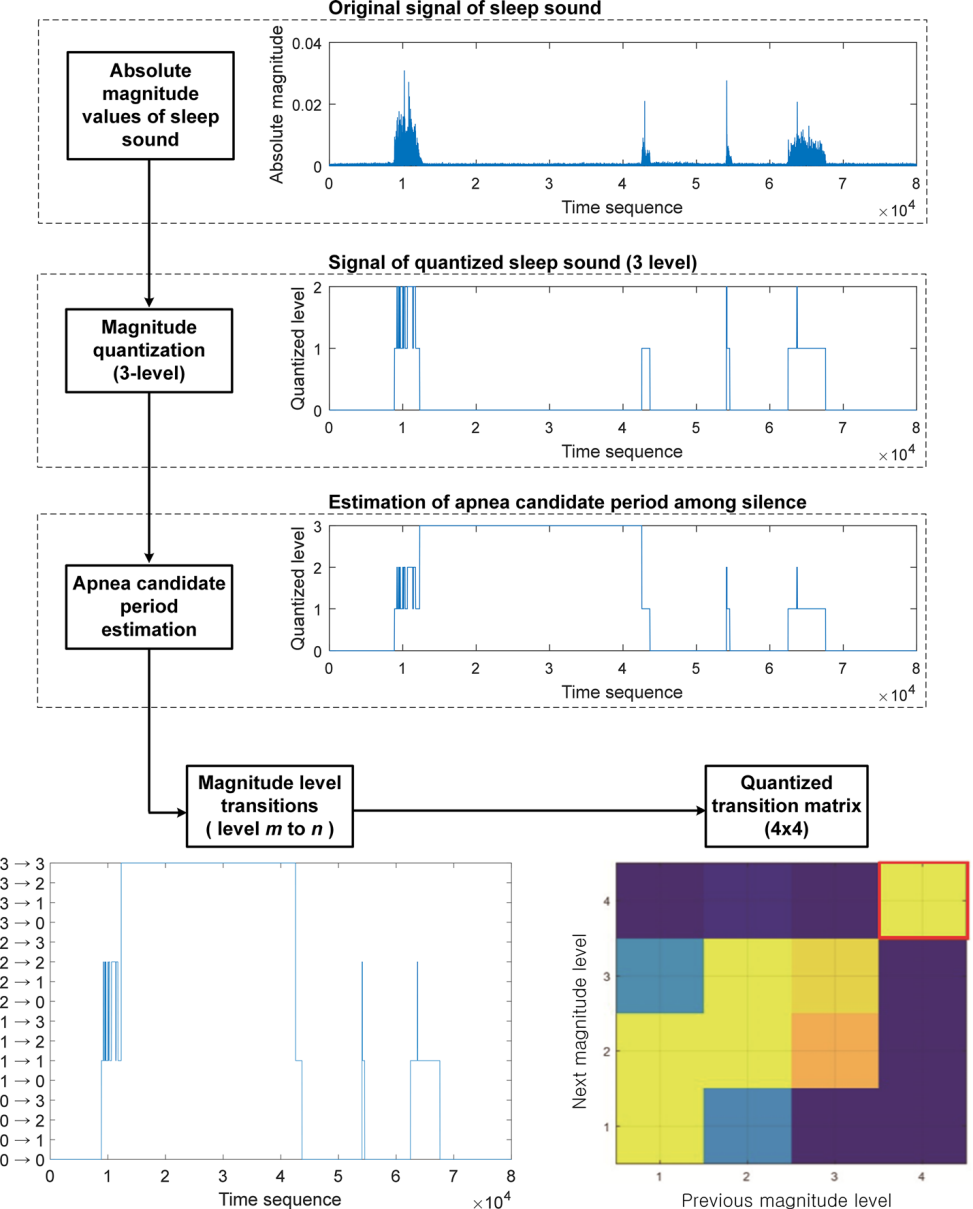
Process of qTM extraction. First, absoluted magnitude values are quantized into three levels (silence, low-level signal, high-level signal) for simplification. Among silence periods, apnea candidate periods were determined under standards of AASM and finally signals were quantized into four levels. Temporal transitions of quantized magnitudes were derived and transition probabilities were calculated over whole sleep

We adopted first derivatives of audio features to reflect temporal changes of sleep sound. However, these may not directly indicate the changes in the magnitude of sound. To overcome this issue, we imported quantized transition matrix (qTM) showing a simplified distribution of transition patterns of the signal magnitude. Figure 4 shows the overall process and an example of qTM extraction. First, for simplification, the absolute magnitudes of signals are quantized into several levels. In this paper, the magnitudes were quantized into three levels, i.e., silence, low-level signal, and high-level signal.

Zero-magnitude (silence) signals can be caused by either apnea or quiet breathing. Discriminating the cause of zero-magnitude signal is important since apnea and quiet breathing differentially affect the severity of sleep breathing disorder. According to the American Association of Sleep Medicine (AASM), episodes of breathing cessation lasting longer than 20 s are considered as obstructive apnea. If a zero-magnitude signal continues for 20–60 s, it is classified as apnea-candidate period and classified as level 4. Otherwise, the signal remains classified as level 0 (silence).

Last, we defined the transition matrix *Q* representing the probability distribution with the size of *m* × *n*, which contains the component *q*_*mn*_ representing the probability of transition from the magnitude level *m* to the level *n* at the subsequent time point. By using the components of transition matrix *Q,* temporal changes in the signals can be easily observed, and *Q* is denominated as qTM.

### Evaluation of the acoustic biomarker

The effectiveness of the defined acoustic biomarker was verified using a classification scenario involving the deep neural network learning technique. A tenfold cross-validation method was adopted for the classification experiments. For every iteration, the patients were disjointly assigned to a training or test group in the ratio of 9:1. The acoustic biomarkers were extracted from every patient in the training group and used to train the model according to the subjects’ SDB severity groupings using three machine learning techniques: simple logistics, support vector machine (SVM), and deep neural network. The SDB severity group of each subject in the test group was then predicted based on their acoustic biomarkers.

**Fig. 5:**
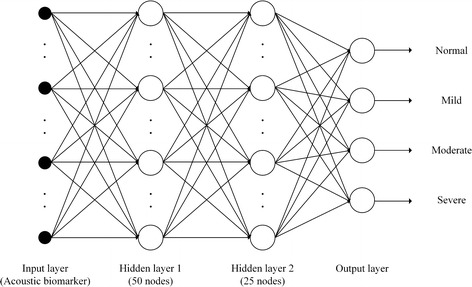
Structure of the deep neural network. The network contains two hidden layers with 50 and 25 nodes respectively, two dropout layers, and an output layer with 4 nodes for 4-class classification

In case of the SVM, polykernel was adopted with an exponent of 1.0 and a c of 1.0. The structure of the deep neural network is described in Fig. 5. The network contained two hidden layers with 50 and 25 nodes respectively, two dropout layers, and an output layer with 4 nodes for 4-class classification. Rectified linear unit (ReLU) was adopted as an activation function. All output values of hidden layers were calculated with the ReLU and used as input values of the next layer. Between hidden layers and the output layer, the dropout technique was adopted to avoid the so-called overfitting problem. Randomly selected 20% of the nodes were eliminated through dropout processes.

Four-group classification tasks were conducted. The classification aimed to predict the AHI severity group of each subject in the test group. The patients were assigned the most suitable severity group based on the extracted acoustic biomarker.

## Results

### Polysomnographic findings

The subjects were 80 men and 40 women, with a mean age of 50.7 (SD 15.7) years. The mean AHI was 22.4 (SD 23.8)/h, and the mean body mass index was 25.4 (SD 4.0) kg/m^2^. General characteristics of each group are shown in Table 2. As shown in Table 2, we conducted ANOVA for each category and found significant differences in the body mass index and the AHI between the groups (p < 0.05). As the age difference of the subject did not show a significant difference according to AHI at the significance level of 0.05, four severity levels were classified based on AHI than age, and in reality, it is general to divide into four severity levels according to AHI.

**Table 2:** Clinical statistics of the population for each group (N = 120)

	Normal	Mild	Moderate	Severe	p value
Age	44.1 ± 20.5	54.8 ± 14.4	53.9 ± 13.3	50.3 ± 16.7	0.0543
Body mass index	23.0 ± 3.9	24.4 ± 3.3	26.9 ± 3.2	27.3 ± 4.19	1.31e−5*
Apnea–hypopnea index	1.3 ± 1.3	9.1 ± 2.6	22.1 ± 4.3	57.5 ± 19.7	1.91e−43*

Mean ± standard deviation, * *p* < 0.05

### Statistical evaluation of audio features

A total of 20 audio features frequently used in the MIR field were extracted (Table 1). Most of the features were composed of a single variable. However, four audio features had multiple variables: the MFCC, linear predictive coding, area method of moments, and area method of moments of MFCC. Forty-eight average values and 110 standard deviation values of audio features were statistically significant. In particular, the p values of 120 statistically significant values were found to be less than 0.001, which is much smaller than the reference value of 0.05, indicating that many audio features differed significantly among the AHI severity groups. However, in the case of the first derivative of the audio features, only 41 values were determined to be significant. Therefore, a small number of values among the significant features was indicative of the change with time.

The F1, F2, and F3 formants were extracted from each analysis window, and several statistical measures were calculated across the windows: the maximum value, minimum value, mean, and standard deviation. Among statistical values, only standard deviation of F1 was determined as significant; however, according to the results of Tukey HSD test, the standard deviation of F1 was not significant for every pair of severity groups.

For sub-band energy distribution, we subdivided the frequency band ranging from 0 to 4000 Hz into eight sub-bands of 500 Hz bandwidth (named sb1, sb1,…,sb8). We derived a Gammatonegram with Gammatone filter bank and calculated simple energy distribution statistics for each sub-band: maximum value, minimum value, mean, and standard deviation, as in the formant analysis. Although many features were revealed as significant by the ANOVA test, most of these were not significant according to Tukey HSD test. Only a few features (standard deviations of sb1, sb2, sb3, sb4, sb5, sb6, sb7, sb8, and minimum values of sb6, sb8) in sub-band analysis could be used as normal discriminators.

For weighted sound intensity, we extracted dBA, dBC, dB and their peak pressure values for every window. The statistical features presented in the two examples above were calculated. We found that the results were similar to those of sub-band analysis. Only standard deviations could be used as discriminators since only these features were revealed as significant by the Tukey-HSD test.

### SDB severity group discriminators

A total of 132 features were identified as group discriminators (62 normal discriminators, 7 moderate discriminators, 63 severe discriminators), as listed in Table 3. No feature was found to discriminate the mild group from the other groups. Final discriminators comprised of general audio features and weighted sound intensity features. In addition, many first derivatives were also shown to be effective, implying the importance of temporal changes in breathing sounds. A set of combined SDB severity group discriminators was regarded as the final acoustic biomarker, which contained 98 audio features collected by taking the ensemble of the discriminators from each group.

**Table 3:** List of SDB severity group discriminators

	Raw features	First derivatives
Normal group discriminator		
Mean	Compactness, FLEW, RDF, AMoM of LCQT 2,7-9	–
Standard deviation	Compactness, MFCC 0,3-11, LPC 6-7, AMoM of MFCC 0-1,4,6, AMoM of LCQT 0,2,4,7-9, AMoM of CQ-MFCC 2,4,7, sb 1-8, A weighted, C weighted, L weighted, peak DB, peak DBA, peak DBC	Compactness, FLEW, MFCC 0,2-3,5-12
Minimum	sb6, sb8	–
Mild group discriminator		
Mean	–	–
Standard deviation	–	–
Moderate group discriminator		
Mean	–	–
Standard deviation	FLEW, MFCC 1	ZC, SF-ZC, MFCC 1, LPC 2,5
Severe group discriminator		
Mean	SF, MM 0, AMoM 0,3,7, AMoM of MFCC 3, AMoM of LCQT 2-3,7-9, AMoM of CQ-MFCC 3,5,9	
Standard deviation	SF, SV, RMS, ZC, SF-ZC, SF-FFT, LPC 1, MM 0, RDF, AMoM 1,3,6-8, AMoM of MFCC 0,1,3,4,6, AMoM of LCQT 0-4, 6-9, AMoM of CQ-MFCC 1-4,6,7, A_weighted, C_weighted, L_weighted, peak_DB, peak_DBA, peak_DBC	SF, SV, RMS, SF-FFT, LPC 1,7, MM 0,2, RDF

Abbreviations of features are listed on Table 1

**Fig. 6:**
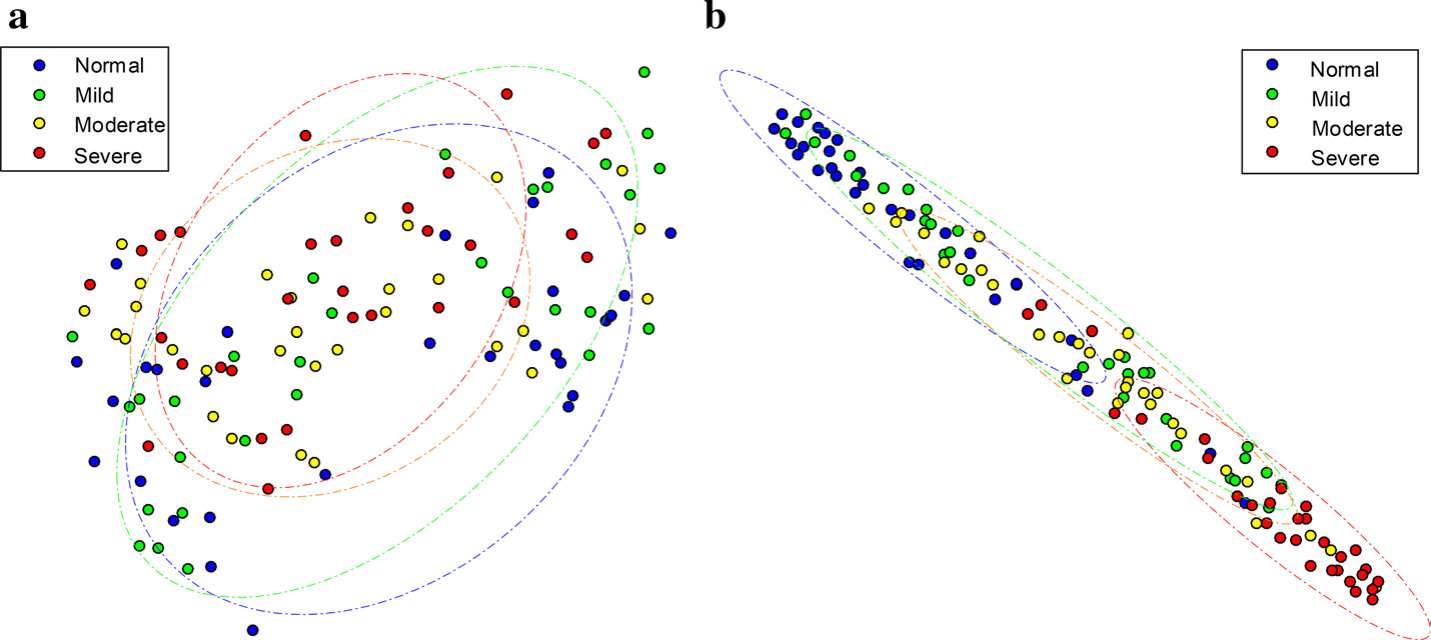
Distribution of subject groups. Using the t-SNE algorithm, distributions of 120 subjects using **a** whole audio features and **b** discriminators (acoustic biomarker) respectively. When using the acoustic biomarker, group separability was increased and it has a direct effect on the classification performance

To visually inspect the effectiveness of the discriminators, we applied t-SNE, a widely-used algorithm for dimension reduction and visualization [29], and projected the subjects onto a two-dimensional plane. Figure 6 illustrates the distribution of 120 subjects when using whole audio features (Fig. 6a) or only the discriminators (Fig. 6b). Dashed circles represent the confidence ellipse, the region that contains 76% of the samples that can be drawn from the underlying Gaussian distribution. As clearly shown in the figure, the overlaps between the groups become much smaller when using the discriminators as compared to whole features. This group separability has a direct effect on the classification performance, as described in the following section.

### Classification experiments using the acoustic biomarker

In order to investigate the effect of each technology element presented in this paper, the baseline was obtained by inputting the statistical values of all extracted audio features and performing four-group classification using the simple logistics classifier. After that, we investigated the change in performance by adding the selected acoustic biomarker and extracted qTM, and compared the results obtained through various classifiers. Finally, the performance of binary classification was compared under various thresholds, and the applicability to screening test and mobile devices examined.

#### Classification results using all statistical values of audio features

**Fig. 7:**
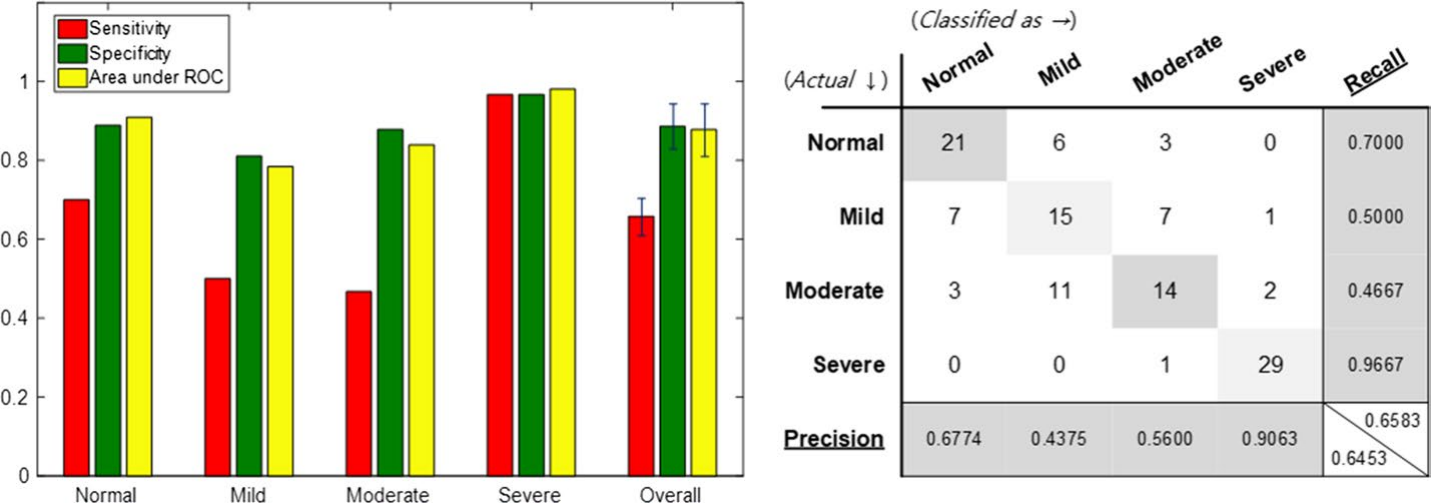
Performance of classification using all audio features (baseline). Specificity, sensitivity and area under ROC curve are depicted when all audio features are adopted as input features. A confusion matrix of the 4-group classification is also presented

Figure 7 shows classification performance when the statistical values of all audio features extracted from the sleep sound are used. Based on the four-group classification, the classification accuracy was of about 65.8%, and the classification accuracy of the normal and severe groups was particularly high. In the case of Mild and Moderate group, we observed that classification accuracy was relatively low. Confusion matrix showed that classification error between the two groups was large.

#### Effect of the window size

**Fig. 8:**
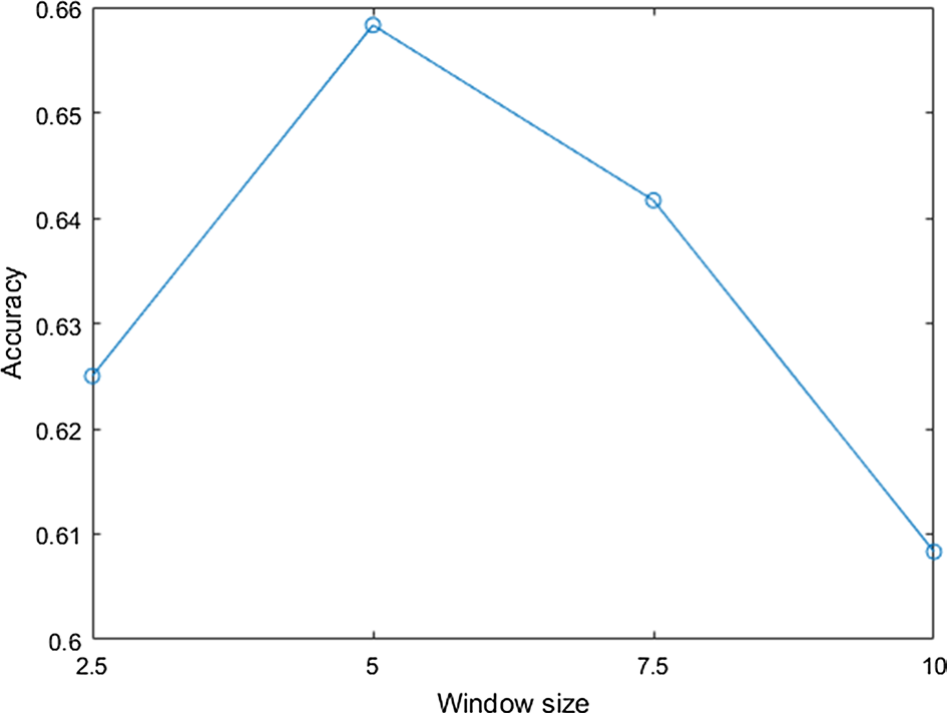
Comparison of performance of using audio features extracted under various window sizes

We adopted four window sizes (2.5, 5, 7.5 and 10 s) when extracting audio features from sleep sounds. Figure 8 shows classification performances using all audio features extracted from segmented sleep sounds according to the window sizes. The graph is concave and the window size of 5 s shows the highest performance of all. Since statistics of audio features are used for learning and the periods of sleep events are multiple seconds in general, fine segmentation is needless and relatively large window size is adequate in our study.

#### Effect of the qTM

**Fig. 9:**
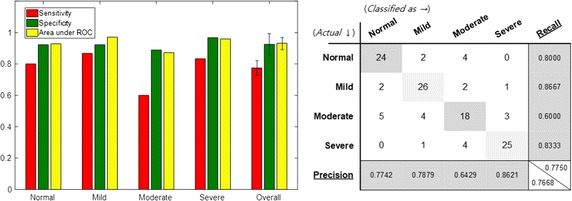
Performance of classification when components of qTM are added as input features

Classification performance shown in Fig. 9 was obtained when the features representing the temporal change in the sleep sound were added by learning using both the statistical values of all audio features and the qTM extracted from the sleep sound. In general, the accuracy of classification improved in all severity groups. Especially, classification performance of mild group was increased to the level of normal and severe groups. Notably, the performance of the ROC area rose to over 90%.

#### Effect of the acoustic biomarker

**Fig. 10:**
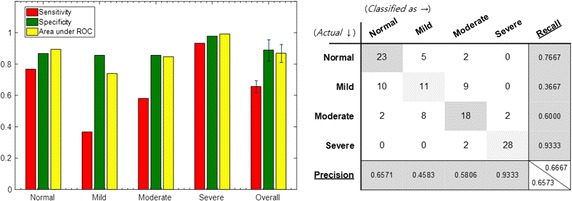
Performance of classification when the acoustic biomarkers are adopted instead of all audio features

**Fig. 11:**
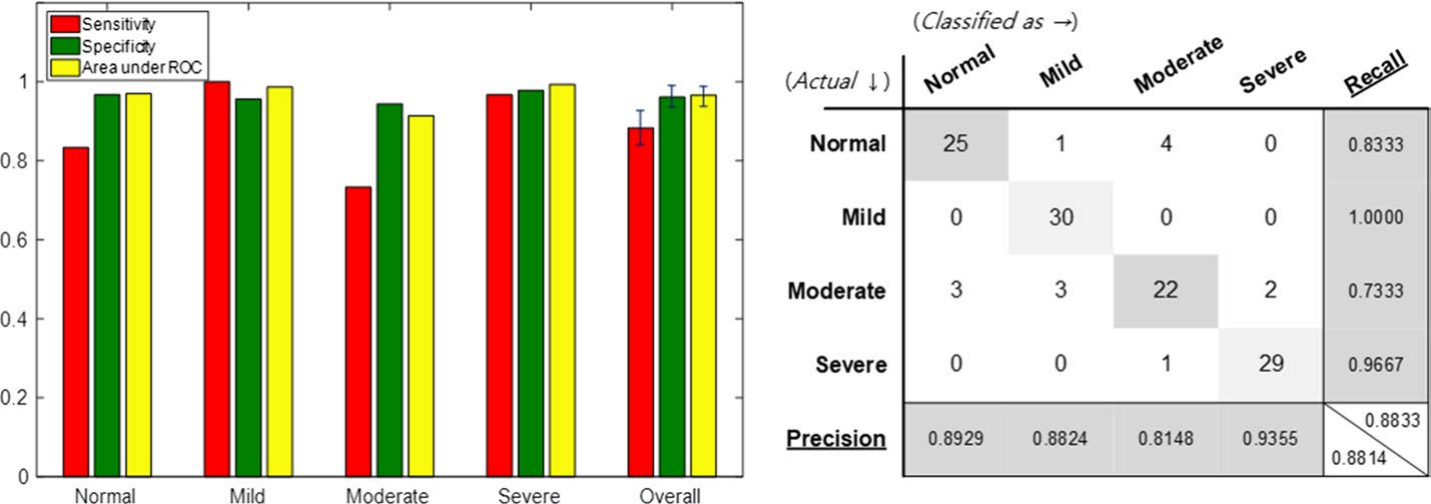
Performance of classification when both the acoustic biomarker and the qTM are adopted

The performance shown in Fig. 10 was obtained when only the feature that distinguishes between the severity groups was selected by acoustic biomarkers and used for learning. In addition, when acoustic biomarkers and qTMs were used for learning, the resulting performance was as the one shown in Fig. 11. The acoustic biomarker alone showed a 66.7% increase in performance, even though the size of input features was reduced to one-third. However, the classification performance of mild and moderate groups was still low, but the addition of the qTM increased the classification performance to 88.3% due to synergy effect. Finally, the use of both acoustic biomarkers and qTMs shows the best performance, and the number of features employed is significantly reduced.

#### Comparison of the results obtained using various classifiers

**Fig. 12:**
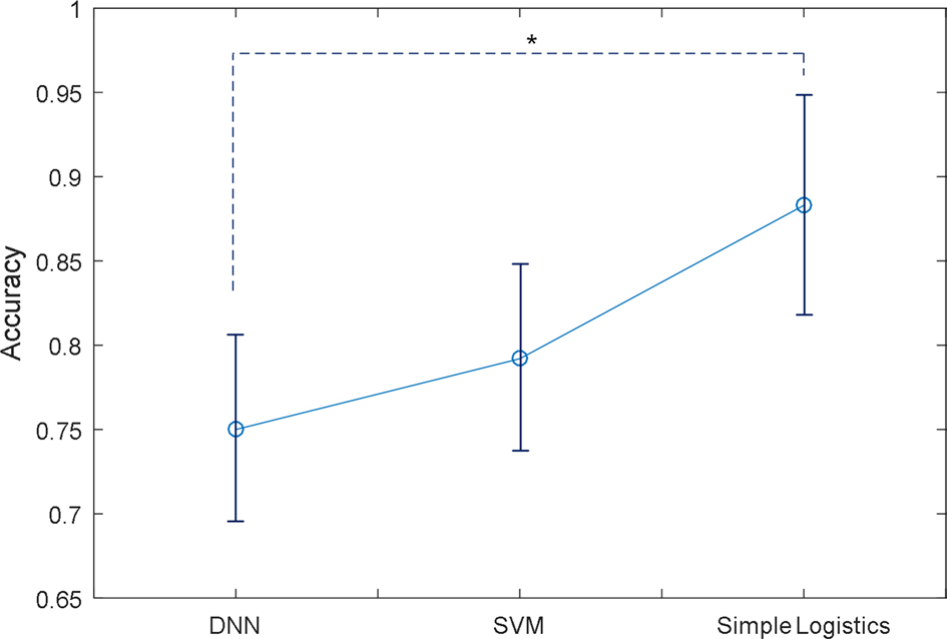
Comparison of performance of using various classifiers

In this section, we compare the results of learning using the three classifiers mentioned in the previous chapter to establish which classifier is suitable. Learning was performed using simple logistics, SVM, and deep neural networks with two hidden layers. It was assumed that both the acoustic biomarker and the qTM were used as input features, and the results of the four-group classification using the above-mentioned three classifiers were compared. The results of the comparison are shown in Fig. 12. Simple logistics showed the best performance and the deep neural network showed low performance due to the small dataset and input features.

#### Effect of SVM-based feature selection on acoustic biomarker

**Fig. 13:**
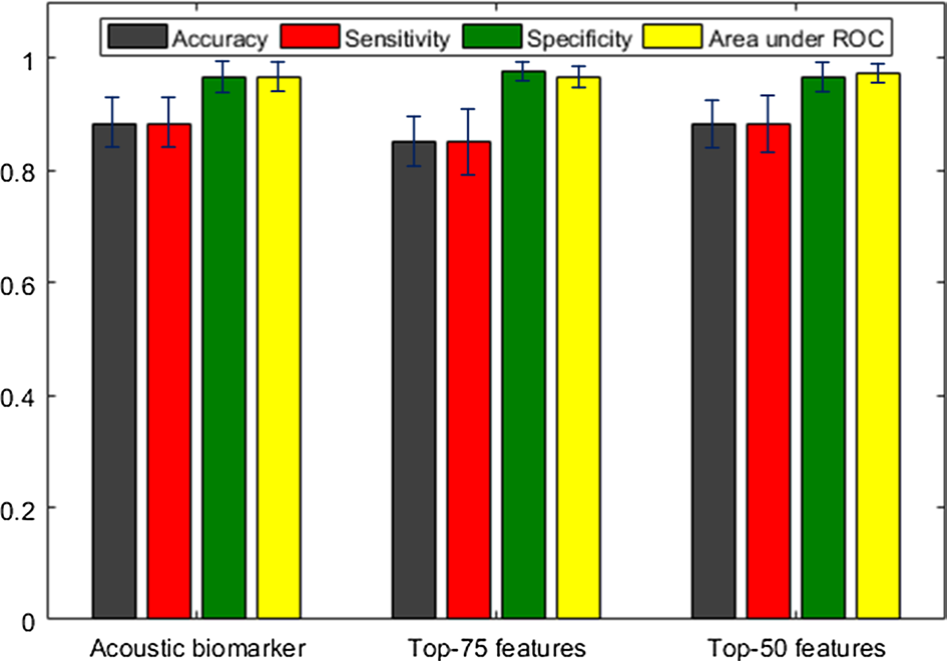
Comparison of performance of using different feature sets chosen with SVM-based feature selection algorithm

We additionally adopted SVM-based feature selection and chose top-50, 75 ranked features. Figure 13 shows performances when all final features, top-75 features and top-50 features are used for classification respectively. We used SVMAttributeEval function in the Weka software [29] for the feature selection. We can find out that there is no difference between performances. However, when we adopt chosen features as input, it may be preferable in terms of the reduction of computational power and time required for the algorithm.

#### Binary classification using multiple thresholds

**Fig. 14:**
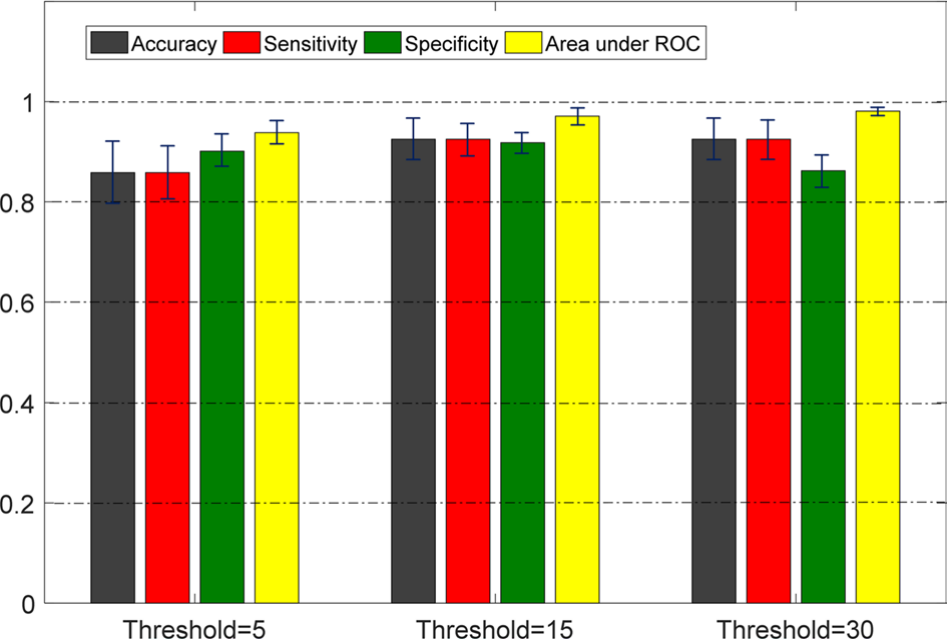
Performance of binary classifications under various thresholds

Unlike the four-group classification, the binary classification divides the patients into two groups according to whether the patient’s AHI value is lower than a certain threshold or not. The results of binary classifications are shown in Fig. 14. The thresholds were set at 5, 15, and 30, which are the boundaries of the AHI range in each severity group. The acoustic biomarker and qTM were used as input features, and the experiments were performed using simple logistics, which was shown to be the best classifier. The accuracy of 92.5% was achieved under the thresholds 15 and 30. In addition, the sensitivity, specificity, and area under ROC were near 90% under all conditions.

## Discussion

The extracted audio features used in this study successfully represented signal patterns in the temporal and spectral domains. Respiratory events during sleep influence the power of the signal of sleep breathing sounds, and many of the extracted audio features illustrate this phenomenon. Certain features also contain several statistical measures of audio signal samples from both domains. Therefore, the audio features can easily represent the great majority of useful traits in the original audio signals, without requiring the use of the whole signal. Moreover, the first derivatives of each feature were also calculated to observe temporal changes in the audio signal. Along with other features, means and standard deviations of the derivatives were calculated, and used as candidate features for the acoustic biomarker. However, not all the extracted features were useful for generating acoustic biomarkers for each subject, as some of extracted features showed no significant differences among the different patient severity groups. It was therefore necessary to verify whether each feature was useful for classifying the severity of SDB.

As shown by the classification task result, use of only the selected acoustic biomarker appeared more advantageous in terms of performance and computation speed than the use of statistical values of all audio features. In addition, when using both the acoustic biomarker and the qTM, the performance was greatly improved because, as the first derivative of the audio feature was generally not statistically significant, the acoustic biomarker did not contain enough elements to represent the temporal change in the sleep sound. Regarding the qTM, this feature indicates the temporal change of the signal, and a significant increase in performance demonstrates that the temporal variation of the signal plays an important role in the prediction of SDB severity.

Although the ANOVA test indicated a significance of various features, it remained difficult to make conclusions regarding the discriminatory power of candidate features. The final determination of acoustic biomarker components was therefore performed based on the results of Tukey HSD test. After determining the acoustic biomarker, a simple classification task was conducted to validate its effectiveness. The means of the features constitute a relatively small portion of all discriminators, suggesting that standard deviations of the features may, in general, be more effective as discriminators. Moreover, since breathing sounds during sleep have time-sequential patterns, many means and standard deviations of the first derivatives could also be used as discriminators.

In our study, both specificity and sensitivity were as high as the accuracy, demonstrating the reliability of the test. While many previous studies conducted binary classifications, it is unusual to evaluate performances using four-group classifications. Another reason for performing four-group classification was to precisely examine the performance. By using both the acoustic biomarker and qTM, it was possible to predict the severity of SDB in subjects with a rather high degree of sensitivity and specificity. This suggests that our model may be adequate for obstructive sleep apnea screening.

Nevertheless, it appears that the deep neural network has a slightly lower classification performance than other classifiers. This is because its learning structure does not satisfy the number of patients or the number of features that can sufficiently learn it, even if it is a simple form with a small number of layers and nodes. In particular, unlike in other studies, this study did not consider snoring events as units of learning. Instead, the features were extracted from the entire sleep surface of the patient and learnt. However, the number of samples was not large enough which is somewhat unsuitable for deep neural network learning. However, if enough sleep sounds are used for learning, the deep neural network will perform better than the results described here.

It should be noted that we conducted a patient-wise classification, while previous studies [4, 30–33] focused on event-wise classifications. Generally, event-wise classification tasks use more training data than patient-wise classification tasks, and thus, derive better results. However, our patient-wise classification still had a higher performance, which proves that the acoustic biomarker effectively represents the characteristics of sleep breathing sounds in patients with SDB.

## Conclusions

We demonstrated that generation of an acoustic biomarker representing the SDB severity score may be used to accurately predict the SDB severity of a patient by analyzing the overnight recording of sleep breathing sound, without conducting the attended full-night PSG.

Simple classification tasks, using extracted features compiled into an acoustic biomarker, were highly performant. This method may be useful for the actual diagnosis because of its high prediction performance, i.e., specificity and accuracy. Therefore, in future, subjects might be able to perform a screening test by themselves with simple devices such as smartphones and smartwatches, using the acoustic biomarker and several machine learning algorithms. Regarding the detection of sleep stages, widely used actigraphy-based sleep stage detection embedded in smartwatches can be adopted in home environment.

Furthermore, our framework can be employed using any recording system since we analyzed sleep sounds recorded by a monitoring video with a low-quality microphone. In addition, the research framework considered in this study may be easily applied to other analogous studies. Improving the quality of recorded sleep breathing sounds may increase the classification performance using the acoustic biomarker.

A more precise prediction model may be derived with more extensive PSG data, since deep neural network is more suitable for learning large scale-data. Thus, additional PSG data should be acquired in the future to increase the diagnostic accuracy of the model.

Additional standard or preprocessed audio features may represent the sleep sound signals, and it would be beneficial to add them as components of the acoustic biomarker. Although temporal change patterns in the audio features within each window are also important in diagnosing SDB, they were not analyzed in this study. Representing these patterns would require identification of additional features. Future work should therefore focus on discovering new features and adding them to the acoustic biomarker to improve the accuracy of classification.

## Abbreviations

SDB: sleep disordered breathing
PSG: polysomnography
AHI: apnea–hypopnea index
SNUBH: Seoul National University Bundang Hospital
REM: rapid eye movement
MFCC: mel frequency cepstrum coefficient
SC: spectral centroid
SR: spectral rolloff point
SF: spectral flux
SV: spectral variability
RMS: root mean square
FLEW: fraction of low energy windows
ZC: zero crossings
SB: strongest beat
BS: beat sum
SSB: strength of strongest beat
CQ: constant-Q
LPC: linear predictive coding
MM: method of moments
RDF: relative difference function
AMoM: area methods of moments
LCQT: log of CQ transform
SPL: sound pressure level
dBA: A-weighted sound pressure level
dBC: C-weighted sound pressure level
dB: linear weighted sound pressure level
HSD: honest significant difference
qTM: quantized transition matrix
AASM: American Association of Sleep Medicine
SVM: support vector machine
ReLU: rectified linear unit
SD: standard deviation
